# Identification of Key Genes and Potential New Biomarkers for Ovarian Aging: A Study Based on RNA-Sequencing Data

**DOI:** 10.3389/fgene.2020.590660

**Published:** 2020-11-16

**Authors:** Lingwei Ma, Huan Lu, Runhua Chen, Meng Wu, Yan Jin, Jinjin Zhang, Shixuan Wang

**Affiliations:** Department of Obstetrics and Gynecology, Tongji Hospital, Tongji Medical College, Huazhong University of Science and Technology, Wuhan, China

**Keywords:** ovarian aging, bioinformatics, GEO database, biomarker, immune cell infiltration

## Abstract

Ovarian aging leads to reproductive and endocrine dysfunction, causing the disorder of multiple organs in the body and even declined quality of offspring’s health. However, few studies have investigated the changes in gene expression profile in the ovarian aging process. Here, we applied integrated bioinformatics to screen, identify, and validate the critical pathogenic genes involved in ovarian aging and uncover potential molecular mechanisms. The expression profiles of GSE84078 were downloaded from the Gene Expression Omnibus (GEO) database, which included the data from ovarian samples of 10 normal C57BL/6 mice, including old (21–22 months old, ovarian failure period) and young (5–6 months old, reproductive bloom period) ovaries. First, we filtered 931 differentially expressed genes (DEGs), including 876 upregulated and 55 downregulated genes through comparison between ovarian expression data from old and young mice. Functional enrichment analysis showed that biological functions of DEGs were primarily immune response regulation, cell–cell adhesion, and phagosome pathway. The most closely related genes among DEGs (*Tyrobp*, *Rac2*, *Cd14*, *Zap70*, *Lcp2*, *Itgb2*, *H2-Ab1*, and *Fcer1g*) were identified by constructing a protein–protein interaction (PPI) network and consequently verified using mRNA and protein quantitative detection. Finally, the immune cell infiltration in the ovarian aging process was also evaluated by applying CIBERSORT, and a correlation analysis between hub genes and immune cell type was also performed. The results suggested that plasma cells and naïve CD4^+^ T cells may participate in ovarian aging. The hub genes were positively correlated with memory B cells, plasma cells, M1 macrophages, Th17 cells, and immature dendritic cells. In conclusion, this study indicates that screening for DEGs and pathways in ovarian aging using bioinformatic analysis could provide potential clues for researchers to unveil the molecular mechanism underlying ovarian aging. These results could be of clinical significance and provide effective molecular targets for the treatment of ovarian aging.

## Introduction

Aging is an inevitable, complex, and detrimental process and is among the most significant known risk factors for most human diseases and diseases including tumors, metabolic syndromes, and decline in female fertility (Hayden, 2015). The progressive decline in the female ovarian function with increasing chronological age is known as ovarian aging, which leads to reproductive and endocrine dysfunction. Ovarian aging is governed by the gradual declines in the quantity and quality of ovarian follicles, finally resulting in menopause, the natural consequence of physiological reproductive aging (Younis, 2012). It is an important issue associated with the health of women and their offspring (Wise et al., 1996). In women, the incidence of cardiovascular disease, osteoporosis, Alzheimer’s disease, obesity, tumors, and diabetes increases with menopause. Moreover, the age-related decline in oocyte quality affects not only the rate of fertilization and development of embryos but also the fate of offspring (Cimadomo et al., 2018). Thus, ovarian aging is regarded as the pacemaker of female aging in general, as it exhibits a higher rate of aging than other organs (Couzin-Frankel, 2013). Accordingly, it is necessary to study and identify the changes in gene expression profiles of aging ovaries to facilitate the early assessment and intervention of ovarian aging.

Several studies indicate that multiple genes affect the development of the ovarian reserve and dynamics of ovarian follicles (Pelosi et al., 2015), and regulation of gene expression also plays an essential role in ovarian aging. Stolk et al. (2009) reported that six SNPs in three loci at Chr 13, Chr 19, and Chr 20 are associated with the timing of ovarian aging in European women. Moreover, multiple linkage analysis and genome-wide association studies identified a number of genes involved in steroid pathways (AMH, AMHR2, COMT, CYP1A1, CYP1B1, CYP17A1, CYP19A1, ESR1, ESR2, FSHR, HSD17B1, and SRD5A2) (Gorai et al., 2003; Hefler et al., 2005; Kok et al., 2005; Huber et al., 2006; Long et al., 2006; He et al., 2007; Kevenaar et al., 2007; Mitchell et al., 2008; Zerbetto et al., 2008) and vascular function (AGT, APOE, F II, F VII, F V Leiden, MTHFR, Nos3, and PAI-1) (Worda et al., 2004; Tempfer et al., 2005; van Disseldorp et al., 2008; He et al., 2009; Liu et al., 2010) and other miscellaneous genes (DAZL, HDC, IL-1RA, and VDR) (Riener et al., 2004; Grimm et al., 2005; Zhang et al., 2006; Zerbetto et al., 2008) as candidate genes associated with age at natural menopause; however, the studies showed almost no overlap in the candidate genes identified and very few consistent results (Voorhuis et al., 2010). A few systematic studies have indicated the involvement of specific genetic profile changes and molecular functions (MF) in ovarian aging. Whereas genome-wide association studies mainly focus on identifying unknown loci and single-nucleotide polymorphism in individuals, RNA sequencing (RNA-Seq) performed using the next-generation sequencing technology can provide almost all transcriptional data of specific tissues or organs in certain conditions (Kukurba and Montgomery, 2015). Thus, this efficient and novel method was adopted to measure the gene expression level at a higher resolution.

In the present study, ovarian gene expression profiles of young (5–6 months old) and old (21–22 months old) mice were downloaded from the Gene Expression Omnibus (GEO) database to identify differentially expressed genes (DEGs). The age of the young and old mice corresponds to the age of the female reproductive period (28 years) and menopause (60 years), respectively, in humans. DEGs in the ovaries of old and young ovary mice were screened, and functional enrichment analysis was performed. A protein–protein interaction (PPI) network was constructed using the Search Tool for the Retrieval of Interacting Genes/Proteins (STRING) online database to analyze the associations between DEGs and identify the molecular interactions involved in ovarian aging. The biological functions and key signaling pathways of these DEGs were identified, and the potential protein interaction network was analyzed. Our analysis indicated that immune response regulation played a crucial role in ovarian aging. Therefore, we estimated the proportion of 25 types of immune cells based on the immune gene expression profiles of mice (Chen et al., 2017), which, to the best of our knowledge, has previously not been used in the study of immune cell infiltration in ovarian aging. The immune cell infiltration in the ovaries of old and young mice was evaluated using CIBERSORT, and specific gene-immune cell correlation analysis was also performed. With subsequent experimental verification, our study provided the mechanisms of aging-related genes in ovarian aging and novel targets for delaying or even reversing the aging of the reproductive system.

## Materials and Methods

### Animals and Ethics Statement

Healthy adult female C57BL/6 mice of 6 months old and 1.5 years old used in this study were purchased from the Center for Laboratory Animal Administration of the Center for Disease Control and Prevention of Hubei Province (Wuhan, China). The mice were bred with free access to food and water at a controlled temperature of 25°C under a 12-h light–dark cycle. All of the experimental procedures used for live animal care and handling in this study were approved by the ethics committee of Tongji Hospital, Tongji Medical College, Huazhong University of Science and Technology in China.

### RNA Extraction and Quantitative Real-Time PCR

Total RNA was extracted from ovaries of two different ages using RNAiso plus reagent (Takara, Nojihigashi, Japan). Samples containing 1 μg total RNA were digested with gDNA wiper (Vazyme, Nanjing) and then reverse transcribed into cDNA using HiScript reverse transcriptase (Vazyme, Nanjing). qRT-PCR was performed in triplicate on a CFX96 real-time PCR system (Bio-Rad) at a final volume of 20 μl. The *Gapdh* gene was used as housekeeping control. The mRNA expression of lymphocyte cytosolic protein 2 (*Lcp2*), histocompatibility 2, class II antigen A, beta 1 (*H2-Ab1*), integrin beta 2 (*Itgb2*), TYRO protein tyrosine kinase binding protein (*Tyrobp*), CD74 antigen (*Cd74*), zeta-chain (TCR)-associated protein kinase (*Zap70*), Rac family small GTPase 2 (*Rac2*), CD14 antigen (*Cd14*), protein tyrosine phosphatase, non-receptor type 6 (*Ptpn6*), and *Fcer1g* (Fc receptor, IgE, high affinity I, gamma polypeptide) were detected. All the primer sequences of the above genes are listed in Table 1.

**TABLE 1:** The sequences of primers used in quantitative real-time PCR.

Primer	Primer sequence (5′-3′)
Lcp2	F: AGAATGTCCCGTTTCGCTCAG
	R: TGCTCCTTCTCTCTTCGTTCTT
H2-Ab1	F: AAGGCATTTCGTGTACCAGTTC
	R: CCTCCCGGTTGTAGATGTATCTG
Itgb2	F: CAGGAATGCACCAAGTACAAAGT
	R: GTCACAGCGCAAGGAGTCA
Tyrobp	F: CCCAAGATGCGACTGTTCTTC
	R: GTCCCTTGACCTCGGGAGA
Cd74	F: CGCGACCTCATCTCTAACCAT
	R: ACAGGTTTGGCAGATTTCGGA
Zap70	F: CGTGCGCTTCCACCATTTC
	R: GTTACACGGCTTACGCAGGT
Rac2	F: GACAGTAAGCCGGTGAACCTG
	R: CTGACTAGCGAGAAGCAGATG
Cd14	F: CTCTGTCCTTAAAGCGGCTTAC
	R: GTTGCGGAGGTTCAAGATGTT
Ptpn6	F: GGACTTCTATGACCTGTACGGA
	R: CGAGCAGTTCAGTGGGTACTT
Fcer1g	F: ATCTCAGCCGTGATCTTGTTCT
	R: ACCATACAAAAACAGGACAGCAT
Gapdh	F: GTCCCGTAGACAAAATGGTGA
	R: TGCATTGCTGACAATCTTGAG

### Immunohistochemistry

The ovarian samples of mice were fixed, paraffin embedded, and processed in 5-μm-thick serial sections. After deparaffinization and rehydration through ethanol with gradient concentration (100–75%), the sections were treated with 3% H_2_O_2_ at room temperature and microwaved for 15 min in citrate buffer for antigen retrieval. Sections were incubated with primary anti-rabbit Lcp2 (1:200, Proteintech, China), anti-rabbit H2-Ab1 (1:200, Abclonal, China) antibody at 4°C overnight, and secondary horseradish peroxidase-labeled goat anti-rabbit IgG (SA1022, Boster, Wuhan, China) for 30 min at room temperature. With DAB chromogenic agent (AR1022, Boster, Wuhan, China) and subsequent counterstaining by hematoxylin, the sections were visualized. The images were captured by using the cellSens Dimension software (Olympus Soft Imaging Solutions GmbH; Germany), and the relative expression was analyzed by Image Pro Plus 6.0 software (Media Cybernetics, Silver Spring, MD, United States).

### RNA-Seq Data

The gene expression profiling datasets (GSE84078), computed on the high-throughput sequencing Illumina HiSeq RNA-Seq 2000 platform (GPL13112), were extracted from the study of Schneider et al. (2017) and were downloaded from GEO database^1^. It includes RNA samples from ovaries of five young (5–6 months old) and five old (21–22 months old) mice.

### Data Pre-processing and Screening for DEGs

The data prepared in simple omnibus format in text (SOFT) format were retrieved from the GEO database, calibrated, and normalized via log transformation. The relevant operating instruction code was put into software R (version 3.6.2), and the gene differential expression analysis between old and young group ovarian samples was performed using the limma package (version 3.42.0) (Ritchie et al., 2015)^2^ with fold change >2 and *p* value < 0.05 considered as the threshold. *p* values were calculated with the empirical Bayes method with the trend parameter set to “TRUE.”

### Functional and Pathway Enrichment Analysis

The gene ontology (GO) database^3^ establishes the comprehensive framework for the model of biology, which consists of three items: MF, biological processes (BP), and cellular components (CC) (Gene Ontology Consortium, 2014). Moreover, KEGG is a widely used collection of databases dealing with high-throughput genomes and biological pathways (Kanehisa et al., 2015). GO annotation and KEGG pathway analysis were conducted by “clusterProfiler” (version 3.14.3), an R package for functional classification and enrichment of gene clusters using hypergeometric distribution (Yu et al., 2012). Our pathway analyses were based on KEGG release 95.0.

### Gene Set Enrichment Analysis

Gene set enrichment analysis (GSEA) is a computational method to solve the problem of undetectable, small changes in gene expression by assessing if predefined sets of genes showed statistical significance between different phenotypes (Subramanian et al., 2005). GSEA was performed through the “clusterProfiler” package, and “h.all.v7.1.symbols.gmt” was downloaded as the refence gene set. A false discovery rate (FDR) <0.25 and *P* < 0.05 were considered as the cutoff values.

### Gene Set Variation Analysis

We also performed gene set variation analysis (GSVA) (Hänzelmann et al., 2013) to further explore the pathway activity based on the 50 hallmark pathways described in the molecular signature database (version 7.2) (Subramanian et al., 2005), allowing the evaluation of pathway enrichment for each sample. By using the “limma” package to perform differential expression analysis of the GSVA results, pathways with significant differences between old and young samples can be obtained, which are more biologically meaningful and interpretable than genes analysis.

### PPI Network Construction and Identification of Hub Genes

The STRING database is a biological database and web resource of known and predicted PPIs, which is commonly used to build a PPI network of DEGs (Mering et al., 2003). After setting a cutoff criterion of the minimum required interaction score as 0.700 (medium confidence) and removing the isolated and partially connected nodes, a complex network of DEGs was constructed by utilizing the Cytoscape software (version 3.7.2) as the visualization tool (Shannon et al., 2003). CentiScape2.2 (Scardoni et al., 2009), a Cytoscape plugin for network centralities analysis, is utilized to identify the most relevant nodes. By setting a degree higher than 5, the top 25 were considered as hub nodes.

### Evaluation of Immune Cell Infiltration

The infiltration of 25 types of immune cells was analyzed according to CIBERSORT R script (Chen et al., 2018) and the immune expression profile of mouse tissues (Chen et al., 2017). The cell composition was visualized by “ggplot2” and “pheatmap” packages. Spearman correlation analysis on the hub genes with high scores (*Lcp2* and *H2-Ab1*) and infiltrating immune cells was performed. The results of the relationship between genes and specific cell type were visualized by “ggscatterstats” package.

### Statistical Analysis

Data are expressed as means ± SD. Unpaired Student’s *t* test was performed. *p* < 0.05 was considered statistically significant. Statistical analysis of all data was processed using GraphPad Prism 7.0 (GraphPad Software, San Diego, CA, United States).

## Results

### RNA-Seq Data and Identification of DEGs in Aging Mouse Ovaries

**FIGURE 1:**
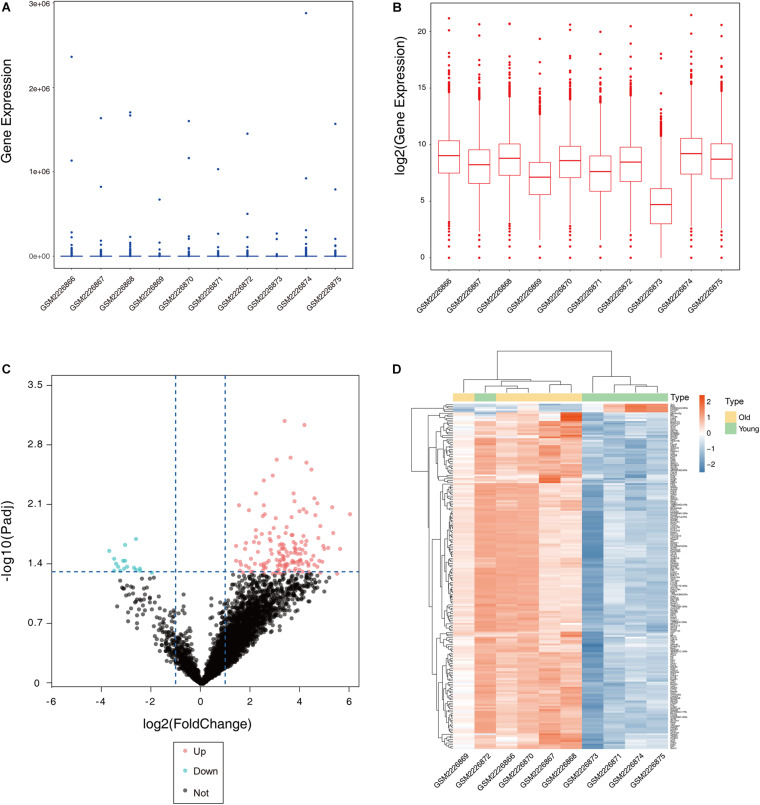
Boxplot, volcano plot, and heatmap of gene expression of the dataset GSE84078. Box and whisker plot of GSE84078 before normalization **(A)** and after normalization **(B)**. Volcano plot of old and young groups **(C)**. Heatmap for selected differentially expressed genes between old and young ovary groups. A total of 931 DEGs are screened in normal old and young mice ovaries. A process of gradual color change from red to blue indicates expression values changing from high to low **(D)**. DEGs, differentially expressed genes; FC, fold change.

After the dataset was normalized (Figures 1A,B) and screened using the limma package (corrected *p* value < 0.05, fold change >2), 931 genes were found to be differentially expressed between the two groups (Supplementary Table S1), of which 876 genes were upregulated and 55 genes were downregulated in the ovaries of old mice compared with their expression in the ovaries of young mice. The differential expression of multiple genes in the dataset is shown in Figure 1C. Additionally, a heatmap of the top 50 upregulated and downregulated genes is shown in Figure 1D.

### Functional Annotation and Pathway Enrichment Analysis of DEGs

**FIGURE 2:**
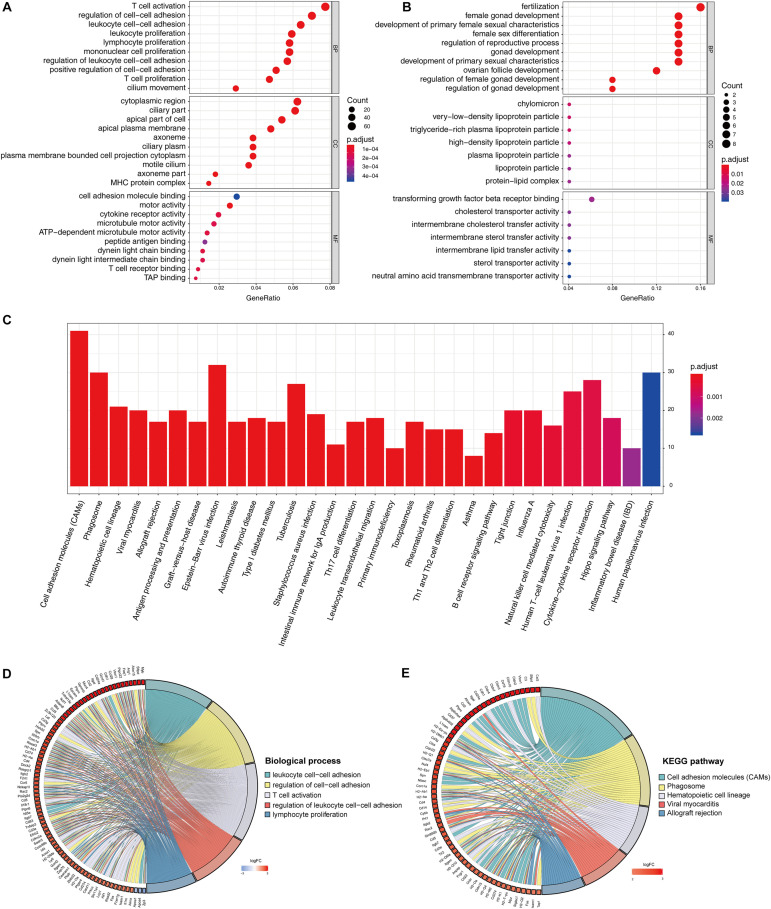
Functional enrichment analysis of DEG. GO enrichment analysis of upregulated DEG in ovarian aging **(A)**. GO enrichment analysis of downregulated DEG in ovarian aging. GO analysis divided DEG into three functional groups: biological process (BP), cellular component (CC), and molecular function (MF) **(B)**. KEGG pathway enrichment analysis of DEG **(C)**. Distribution of DEG in ovarian aging in the significant GO terms of biological process **(D)**. Chord plot of the relationship between DEG and KEGG pathways **(E)**.

The identified DEGs in the ovaries of old and young mice were further analyzed via GO and KEGG pathway analysis using the “clusterProfiler” package (Yu et al., 2012). The GO enrichment analysis classified the DEGs into three functional groups, including biological process, cellular component, and molecular function (Gene Ontology Consortium, 2014). As shown in Figure 2A and Table 2, a high number of upregulated genes were significantly enriched in T cell activation, cell–cell adhesion, and cell projection assembly in the category biological process; ciliary part, cytoplasmic region, and apical part of cell in the category cellular component; and DNA-binding transcription activator activity and RNA polymerase II-specific in the molecular function category. Moreover, the downregulated genes (Figure 2B and Table 3) were highly enriched in fertilization, female gonad development, and female sexual characteristics of development in the category biological process; chylomicron, very-low-density lipoprotein particle, and triglyceride-rich plasma lipoprotein particle in the category cellular component; and transforming growth factor beta receptor binding and cholesterol transporter activity in the category molecular function. Furthermore, the KEGG pathway analysis (Figure 2C) revealed that the most significantly enriched pathways of the upregulated genes were related to cell adhesion molecules (CAMs) (*p* = 7.49E–21) and phagosome (*p* = 3.83E–11), whereas the downregulated genes were involved in vitamin digestion and absorption (*p* = 0.000657) (Supplementary Table S2).

**TABLE 2:** Functional enrichment analysis of upregulated genes in the old mouse ovary compared to young mouse.

Category	Term	Description	Count	%	*p* value
BP	GO:0007159	Leukocyte cell–cell adhesion	53	6.4%	5.35E–21
BP	GO:0022407	Regulation of cell–cell adhesion	58	7.0%	3.23E–20
BP	GO:0042110	T cell activation	64	7.7%	2.21E–19
BP	GO:1903037	Regulation of leukocyte cell–cell adhesion	47	5.7%	1.71E–18
BP	GO:0046651	Lymphocyte proliferation	48	5.8%	5.15E–18
CC	GO:0005930	Axoneme	31	3.7%	7.76E–18
CC	GO:0097014	Ciliary plasm	31	3.7%	9.92E–18
CC	GO:0032838	Plasma membrane bounded cell projection cytoplasm	31	3.7%	3.48E–14
CC	GO:0044441	Ciliary part	50	6.0%	1.27E–12
CC	GO:0044447	Axoneme part	15	1.8%	1.83E–12
MF	GO:0003774	Motor activity	21	2.6%	8.49E–09
MF	GO:0004896	Cytokine receptor activity	16	2.0%	3.34E–07
MF	GO:0045503	Dynein light-chain binding	9	1.1%	4.88E–07
MF	GO:1990939	ATP-dependent microtubule motor activity	11	1.4%	6.23E–07
MF	GO:0042608	T cell receptor binding	7	0.9%	6.35E–07
KEGG	mmu04514	Cell adhesion molecules (CAMs)	41	11.5%	7.49E–21
KEGG	mmu04145	Phagosome	30	8.4%	3.83E–11
KEGG	mmu04640	Hematopoietic cell lineage	21	5.9%	1.65E–10
KEGG	mmu05416	Viral myocarditis	20	5.6%	2.68E–10
KEGG	mmu05330	Allograft rejection	17	4.8%	3.39E–10

**TABLE 3:** Functional enrichment analysis of downregulated genes in the old mouse ovary compared to young mouse.

Category	Term	Description	Count	%	*p* value
BP	GO:0008585	Female gonad development	7	14.0%	4.56E–09
BP	GO:0009566	Fertilization	8	16.0%	5.48E–09
BP	GO:0046545	Development of primary female sexual characteristics	7	14.0%	5.48E–09
BP	GO:0001541	Ovarian follicle development	6	12.0%	8.00E–09
BP	GO:2000194	Regulation of female gonad development	4	8.0%	2.57E–08
CC	GO:0042627	Chylomicron	2	4.0%	0.00028
CC	GO:0034361	Very-low-density lipoprotein particle	2	4.0%	0.000643
CC	GO:0034385	Triglyceride-rich plasma lipoprotein particle	2	4.0%	0.000643
CC	GO:0034364	High-density lipoprotein particle	2	4.0%	0.001151
CC	GO:0034358	Plasma lipoprotein particle	2	4.0%	0.002444
MF	GO:0005160	Transforming growth factor beta receptor binding	2	4.0%	0.000205
MF	GO:0017127	Cholesterol transporter activity	2	4.0%	0.000844
KEGG	mmu04977	Vitamin digestion and absorption	2	14.3%	0.000657
KEGG	mmu04975	Fat digestion and absorption	2	14.3%	0.001829
KEGG	mmu04979	Cholesterol metabolism	2	14.3%	0.002735

The association of multiple annotation categories and the genes involved in ovarian aging was further investigated using a GO chord plot to visualize the complex connections between the upregulated genes and BP of GO terms (Figure 2D) and KEGG pathways (Figure 2E). Specifically, BP were grouped according to the functional theme presented in Supplementary Figure S1, in which the terms are mainly categorized into four subgroups, namely, cell movement, immune system activation and regulation, cell–cell adhesion, and immune cell proliferation. Overall, the immune system regulation plays a crucial role in ovarian aging.

### PPI Network and Hub Gene Analysis

**FIGURE 3:**
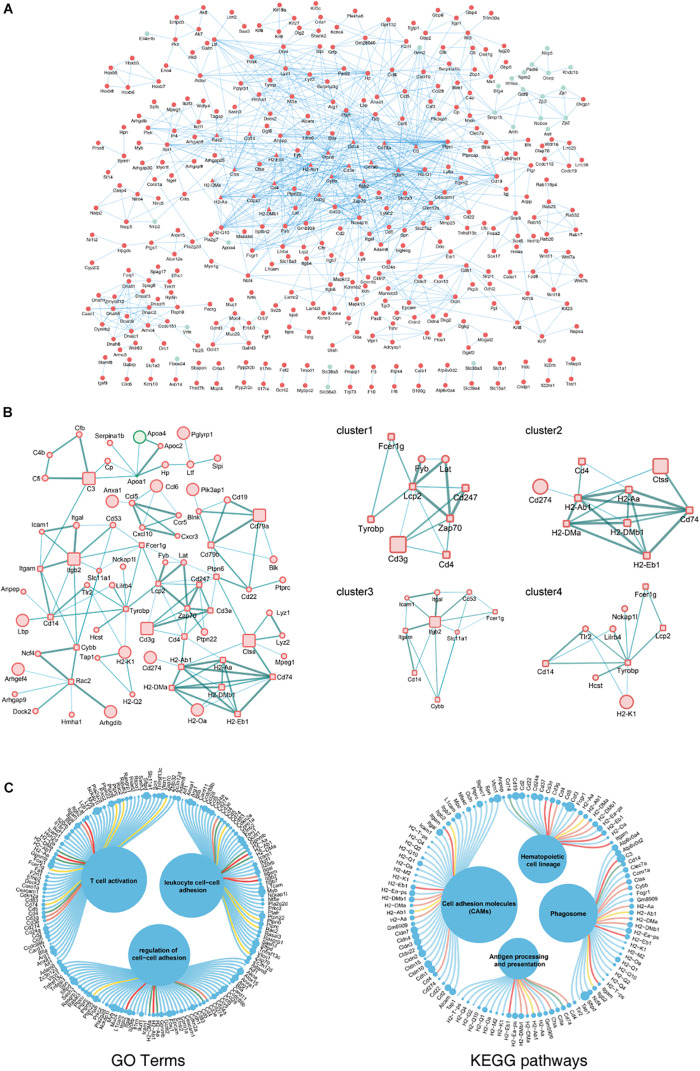
PPI network complex of DEGs in ovarian aging. Red nodes represent upregulated genes; green nodes represent downregulated genes. Round rectangle nodes represent hub genes **(A)**. Four of the highly connected clusters were identified by centrality calculating algorithms and visualized by the Centiscape plugin of Cytoscape. Interactions are color coded according to combined scores with darker edges corresponding to higher scores **(B)**. Plot of hub genes displayed in top GO terms and KEGG pathways. Size of circles represents the relative mRNA expression of the genes **(C)**.

The biological functions of the DEGs identified in aging ovaries were systematically analyzed by constructing a PPI network of DEGs using Cytoscape. In the PPI network with a total of 931 DEGs, 362 nodes and 937 edges were mapped with the minimum required interaction score >0.7 (Figure 3A). By screening PPIs with a score >0.95, hub genes were identified and classified using Centiscape 2.2, a plugin of Cytoscape. The hub genes with the highest connectivity were *Lcp2* (degree = 9), *H2-Ab1* (degree = 8), *Itgb2* (degree = 8), *Tyrobp* (degree = 8), and *Cd74* (degree = 7), as shown in Figure 3B. To obtain a better understanding of the relationship between hub genes and the GO terms and KEGG pathways, an illustration of hub genes in the annotation terms was generated (Figure 3C). These results showed that most hub genes involved in ovarian aging also participated in the BP of T cell activation and regulation of cell–cell adhesions, indicative of biomarkers of ovarian aging.

### GSEA and Gene Set Variation Analysis Revealed Biological Functions of Hub Genes in Ovarian Aging

**FIGURE 4:**
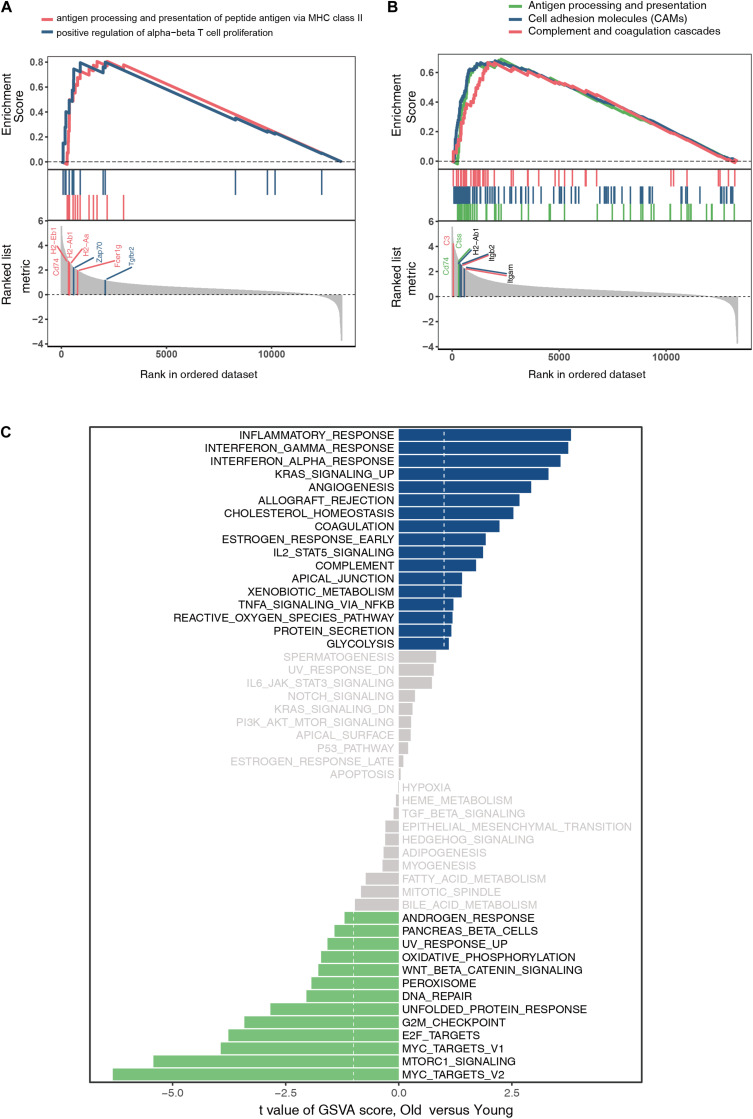
Gene set enrichment analysis of GO terms **(A)**. Gene set enrichment analysis of KEGG pathways **(B)**. Gene set variation analysis and the differentially expressed analysis of pathways **(C)**.

The GO and KEGG analyses of DEGs focus only on the biological functions and pathways, which may always overlook the genes that show non-significant changes in expression. GSEA is a computational method to solve the problem of undetectable, minor changes in gene expression. The results of GSEA indicated that the enriched GO terms were involved in antigen processing and presentation of peptide antigens via major histocompatibility complex (MHC) class II and positive regulation of alpha–beta T cell proliferation (Figure 4A). Similarly, the enriched KEGG pathways were found to be mainly related to antigen processing and presentation, CAMs, and complement and coagulation cascades (Figure 4B). Genes such as *Cd74*, *H2-Ab1*, *H2-Aa*, *Zap70*, *Fcer1g*, *Tgfbr2*, *C3*, *Ctss*, *Itgb2*, and *Itgam* were the top-ranked genes in the GSEA. Additionally, GSVA and differential expression analysis of pathways revealed that inflammatory response, interferon gamma response, interferon alpha response, and reactive oxygen species showed high scores (Figure 4C). These findings suggest that immune response plays an important role in ovarian aging.

### Immune Cell Infiltration and Correlation Analysis

**FIGURE 5:**
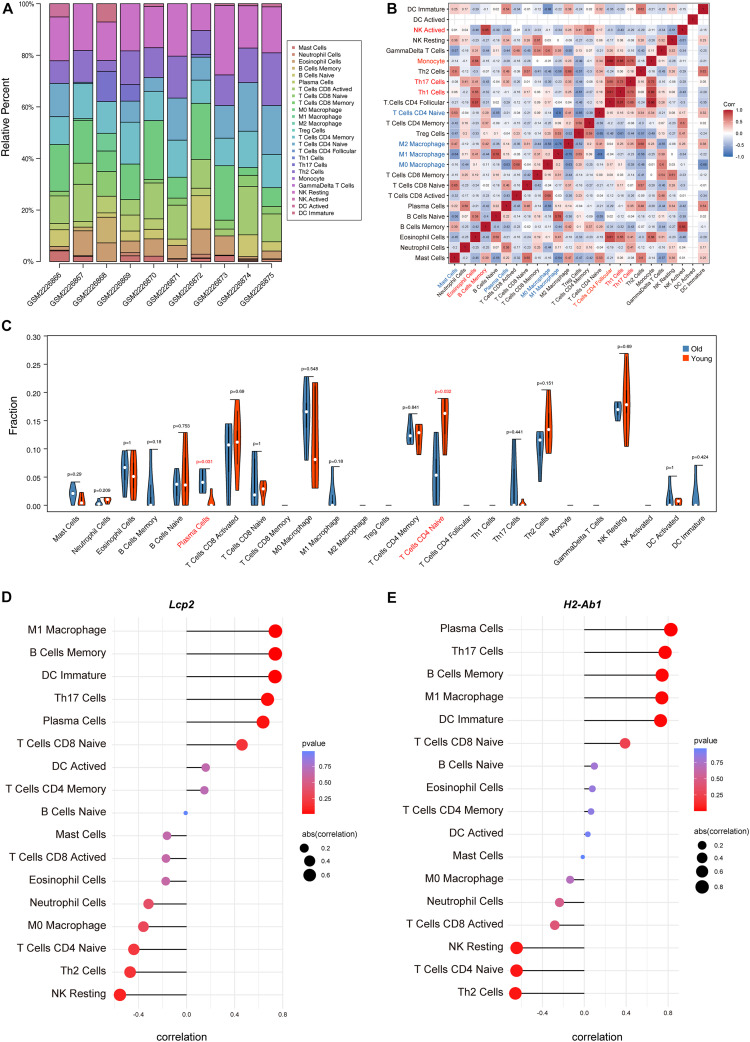
Immune cell infiltration evaluation and correlation analysis. The composition of 25 immune cell types in each sample **(A)**. Correlation heatmap of the immune cell types **(B)**; the intensity of the color indicated the strength of the correlation; red represents a positive correlation, while blue represents a negative correlation; violin plot of the proportion of various immune cell types **(C)**; *p* value < 0.05 was marked red. Correlation between *Lcp2* and infiltrating immune cells **(D)**. Correlation between *H2-Ab1* and infiltrating immune cells **(E)**.

**FIGURE 6:**
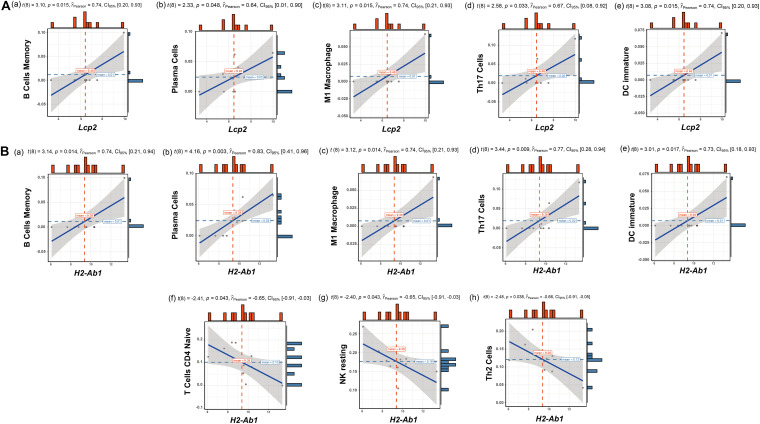
Correlation analysis of *Lcp2*, *H2-Ab1*, and their responding immune cell types. Correlation analysis of *Lcp2* and the listed immune cell types **(A)**: memory B cells (a), plasma cells (b), m1 macrophages (c), Th17 cells (d), and immature dendritic cells (e). Correlation analysis of *H2-Ab1* and the listed immune cell types **(B)**: memory B cells (a), plasma cells (b), m1 macrophages (c), Th17 cells (d), immature dendritic cells (e), naïve CD4^+^ T cells (f), Th2 cells (g), and resting NK cells (h).

The above findings suggest the involvement of various immune-related BP in ovarian aging. To further explore the role of immune cell infiltration in ovarian aging, we quantified 25 types of immune cells according to CIBERSORT by using mouse immune cell gene expression matrix as control. The composition of the immune cell types is presented in Figure 5A. The correlation heatmap (Figure 5B) of the immune cell types indicated that activated natural killer (NK) cells and memory B cells were significantly positively correlated. Monocytes, Th1 cells, CD4^+^ T follicular cells, and eosinophils were also positively correlated. Moreover, Th1 cells, CD4^+^ T follicular cells, and monocytes had a significant positive correlation. M1 macrophages, regulatory T cells, and naïve CD4^+^ T cells were significantly negatively correlated. Next, we compared the proportions of immune cells in the ovaries of old and young mice, and the cell types with extremely low values were excluded. A grouped violin plot showed a significant difference in plasma cells and naïve CD4^+^ T cells between the ovaries of old and young mice; ovaries of old mice showed a higher plasma cell infiltration and lower naïve CD4^+^ T cell infiltration than the ovaries of young mice (Figure 5C). Correlation analysis of the genes and immune cells showed that one of the highly scored hub genes, *Lcp2*, was positively correlated with memory B cells (*r* = 0.738, *p* = 0.015), plasma cells (*r* = 0.64, *p* = 0.047), m1 macrophages (*r* = 0.739, *p* = 0.015), Th17 cells (*r* = 0.674, *p* = 0.033), and immature dendritic cells (*r* = 0.736, *p* = 0.015; Figures 5D**,** 6A). *H2-Ab1* (Figure 5E) was positively correlated with memory B cells (*r* = 0.744, *p* = 0.013), plasma cells (*r* = 0.827, *p* = 0.003), m1 macrophages (*r* = 0.741, *p* = 0.014), Th17 cells (*r* = 0.772, *p* = 0.009), and immature dendritic cells (*r* = 0.729, *p* = 0.016; Figure 6B(a–e)) but negatively correlated with naïve CD4^+^ T cells (*r* = −0.648, *p* = 0.043), Th2 cells (*r* = −0.659, *p* = 0.038), and resting NK cells (*r* = −0.648, *p* = 0.043; Figure 6B(f–h)). The above results provide a more detailed description of the correlation between hub genes and immune cells.

### Expression Levels of the Above Hub Genes in the Ovaries of Young and Old Mice

**FIGURE 7:**
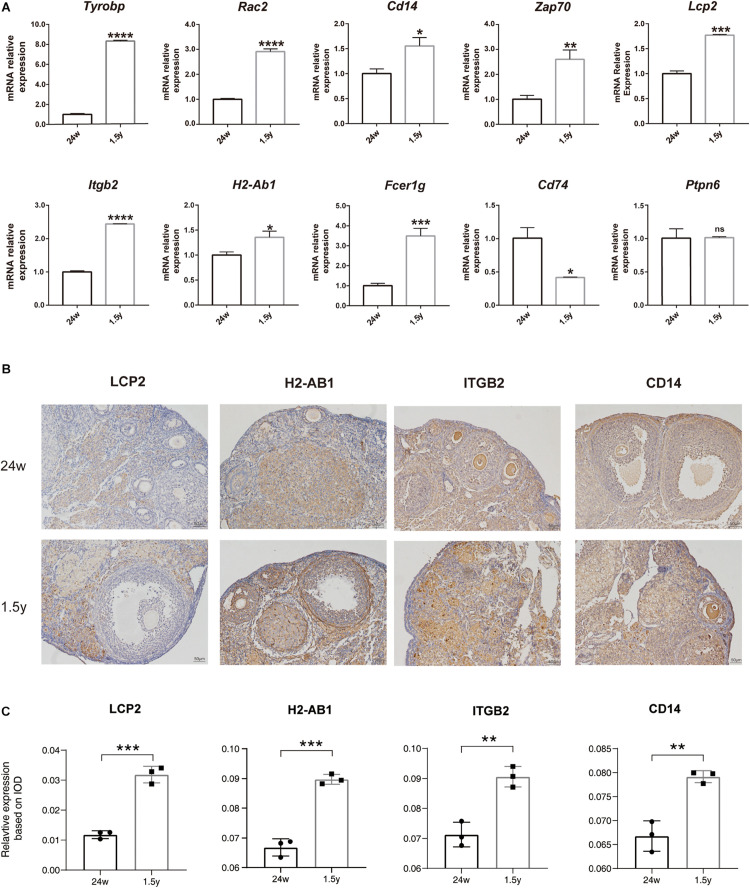
Experiment validation of hub genes with top high degree scores. Relative mRNA expression levels of hub genes expression in the 24-week-old and 1.5-year-old mice ovary (unpaired Student’s *t* test) **(A)**. Representative images of IHC detection of selected hub genes (*Lcp2*, *H2-Ab1*, *Itgb2*, and *CD14*) expression in the 24-week-old and 1.5-year-old mice ovary **(B)**. Relative expression level analysis of LCP2, IGTB2, and CD14 (unpaired Student’s *t* test) **(C)**. *p < 0.05; **p < 0.005; ***p < 0.001; ****p < 0.0001.

To confirm the results of hub gene analysis and investigate the transcriptomics expression patterns associated with ovarian aging, qPCR analysis and immunohistochemistry staining were performed using the ovarian samples from old (1.5 years) and young (6 months) mice (*n* = 5). As shown in Figure 7A, the mRNA expression of *Tyrobp*, *Rac2*, *Cd14*, *Zap70*, *Lcp2*, *Itgb2*, *H2-ab1*, and *Fcer1g* was significantly higher in older ovaries. These genes were involved in immune cell signaling and activation (*Tyrobp*, *Lcp2*, *Itgb2*, and *H2-Ab1*), immune system regulation (*Cd14* and *Zap70*), and reactive oxygen species production (*Rac2*). However, the relative mRNA expression of the hub gene *Ptpn6* showed no significant difference, whereas the relative mRNA expression of *Cd74* showed a reverse tendency. Moreover, the immunohistochemical assay showed that the expression levels of specific antibodies to the hub genes with top high degree scores (*Lcp2*, *H2-Ab1*, *Itgb2*, and *CD14*) in the ovaries of old mice were significantly higher than those in the ovaries from young mice (Figures 7B,C). Overall, the bioinformatic findings of putative hub genes were validated by molecular experiments.

## Discussion

Female reproductive and endocrine function declines with ovarian aging. Ovarian aging accelerates the dysfunction of multiple organs in the body; however, the mechanism of ovarian aging has not been fully elucidated. Therefore, a comprehensive understanding of the gene expression profile associated with ovarian aging is of significant importance. To date, there are little data on the specific mechanisms responsible for ovarian aging.

In the present study, we first analyzed the RNA-Seq data of ovarian samples, young (5–6 months old) and old (21–22 months old) mice, with the ages corresponding to the female reproductive age period (28 years) and menopause (60 years), respectively, in humans. Next, we identified DEGs; conducted GO functional enrichment and KEGG pathway analyses, GSEA, and GSVA; constructed a PPI network; and identified hub genes to investigate the molecular mechanisms underlying ovarian aging and explore therapeutic targets.

Further functional and pathway enrichment analysis showed that the most significant GO terms and KEGG pathways associated with the upregulated DEGs were related to immune response, regulation of cell–cell adhesion, and phagosome. The expression of oocyte- and germline-specific genes decreased with age, which was consistent with the physiological aging process of the female reproductive system. As both GO and KEGG pathway analyses results identified immune-related gene CAMs, they may be an essential part of ovarian aging. The immune system plays an essential role in the regulation of tissue homeostasis, including ovarian homeostasis. Thymectomized 3-day-old mice have been shown to develop ovarian dysgenesis and fertility impairment, which could be rescued by the replacement of thymic and lymphoid tissues (Sakakura and Nishizuka, 1972). Moreover, the immune system declines with aging, followed by the ovarian function, indicating that there is a strong relationship between the immune system and ovaries (Bukovsky and Caudle, 2012). Previous studies indicate that immune cells such as macrophages, neutrophils, and NK cells are found in the tissues of the hypothalamic–pituitary–ovarian axis, and the cytokines (IL-6, IL-1, TGF-β, TNF-α, IL-2, and INF-γ) secreted by them affect hormone production and secretion (Bukovsky and Presl, 1979). Moreover, immune cell-derived cytokines are significant regulators of ovarian function. In a previous study, Sharov et al. (2008) indicated that genes that exhibited significant age-related changes in expression were categorized into genes associated with immune response, including interleukins and interleukin receptors, and MHC II. Furthermore, a series of studies that analyzed the transcriptional profiles of aging emphasized the significance of genes related to inflammation and immune response, which were overexpressed with increasing age (De Magalhães et al., 2009; López-Otín et al., 2013; Jia et al., 2018).

Cell adhesion is the process by which cells interact with and bind to neighboring cells through specialized cell surface molecules. In the vascular system, vascular adhesion molecule-1 (VCAM-1) and carcinoembryonic antigen-related cell adhesion molecule 1 (CEACAM1) were identified to play crucial roles in age-associated vascular alterations, including increased oxidative stress, vascular fibrosis, and endothelial barrier impairment (Zou et al., 2006; Kleefeldt et al., 2019). In the present study, cell–cell adhesion and CAMs were identified in both GO enrichment and KEGG pathway analyses and could therefore be involved in ovarian aging. In mouse ovaries, E-cadherin, a transmembrane protein expressed in the cytomembrane of oocytes in primordial follicles, plays an indispensable role in the maintenance of the primordial follicle pool and female reproductive ability (Yan et al., 2019). Follicular growth initiation is marked by cuboidalization of flattened granulosa cell; adherens junction proteins such as N-cadherin and nectin have been shown to regulate this process (Mora et al., 2012). To date, although there are a significant number of studies on cell adhesion in the context of aging, research on cell adhesion with respect to ovarian aging remains limited. Therefore, we speculate that CAMs, a crucial part of tissue homeostasis, participates in ovarian aging and dysfunction.

Additionally, we performed a PPI network analysis of DEGs and identified hub genes, which were mostly involved in the immune system, that may play a crucial part of ovarian aging. Genes such as *Lcp2*, *H2-ab1*, *Itgb2*, *Tyrobp*, and *Cd74* identified in our study have previously been shown to play a role in aging. *Lcp2* was reported to be involved in kidney aging and to participate in the interferon gamma pathway on the basis of a comparison between kidneys of young (6 months old) and old (24 months old) rats (Shavlakadze et al., 2019). Moreover, *Cx3cr1*-deficient microglia of young mice (2 months old) exhibited a premature aging phenotype; furthermore, a comparison of the wild-type and *Cx3cr1*-deficient microglia of young mice identified DEGs associated with MHC class II (*H2-Ab1*, *H2-Aa*, and *H2-Eb1*). Mukherjee et al. (2019) reported that *Tyrobp* and *Fcer1g* were among the hub genes in the gene network strongly associated with both aging and neurodegenerative diseases. A marked change observed in aging is also related to the composition and functionality of CD4^+^ T cells, the commander of adaptive immune responses. The dynamic reorganization of CD4^+^ T cell subsets were promoted by aging, in which activated regulatory T cells overexpressing Cd74 and other markers were identified (Elyahu et al., 2019).

Moreover, phagosome pathway was also identified in our analysis of ovarian aging. Phagosome pathway is crucial for tissue homeostasis and both innate and adaptive host defense against pathogens and plays an important role in inflammation, antigen presentation, and autophagy. Bodea et al. (2014) reported that in neurodegeneration, the microglial complement–phagosome pathway was activated, and complement *C3*-deficient mice were protected against neurodegeneration in age-dependent synaptic and neuronal loss (Shi et al., 2017). In the analyses mentioned above, several studies on aging identified *Ctss* and *C3* to be among overexpressed genes associated with inflammation.

The hub genes identified in our analysis also played critical roles in the pathways with high scores in the GSVA results. A splice variant of *Lcp2* can reduce the quantity of normal SLP-76 protein by up to 90%, leading to abnormal immunogenic and tolerogenic pathways, which was also verified by the mutant *Lcp2*^twp/twp^ mice phenotype (Siggs et al., 2015). Stables et al. (2011) also reported that compared to macrophages from hyper-inflamed mice with naïve and pro-inflammatory macrophages from the un-inflamed peritoneum, *H2-Ab1* and *Cd74* were enriched for antigen processing/presentation. Deng et al. (2017) found that the genetic defect of *H2-Ab1* in adipocytes can reduce IFNγ and increase Treg content in mice fat, which leads to a decrease in fat inflammation and insulin resistance caused by obesity. In a word, the hub genes screened out in DEGs also contribute to the key pathways such as immune response and inflammatory response (Figure 4C).

From the above results, we concluded that immune response and related BP play a major role in ovarian aging. Hub genes like *Cd47*, *Ctss*, *H2-Ab1*, *Itgb2*, and *Itgam* participated in the antigen processing and presentation pathways and complement and coagulation cascades in GSEA results (Figure 4B), indicating that immune response is a critical event in ovarian aging. Wong and Goldstein (2013) reported that aging impairs the antigen presentation function of dendritic cells and thus could exert adverse impact on the immune system. Moreover, the complement system was also found to be critical in maintaining retinal integrity during aging by using complement knockout mice models (*C1q*^–/–^, *Mbl a/c*^–/–^, *Fb*^–/–^, *C3*^–/–^, and *C5*^–/–^) (Mukai et al., 2018). Thus, we assumed that immune response is one of the major causes of ovarian aging. As to the reproductive system, the abnormal production of autoimmune antibodies due to a viral infection, which targets the ovarian tissue, is the most common reason of ovarian dysfunction (Luborsky et al., 1999). Previous studies on autoimmune ovarian aging have mostly focused on the infiltration of lymphocytes and plasma cells into the ovary, abnormal distribution of T lymphocytes in peripheral blood, and the production of antibodies in oocytes or granulosa cells. Previous studies suggest that patients with premature ovarian failure (POF) demonstrate a decreased CD4^+^/CD8^+^ cell ratio, which is associated with lower serum estradiol levels. The CD4^+^/CD8^+^ cell ratio was also found to be significantly lower in older mice than in younger mice, which suggests immune dysregulation (Shao et al., 2017). Females with POF were demonstrated to have a higher number of activated T cells and B cells, and a lower number of NK cells, indicating that immune disorders are the main cause of POF (Chernyshov et al., 2001). Therefore, it is crucial to assess immune cell infiltration and investigate the correlation between gene expressions and immune cells in ovarian aging. We used CIBERSORT to perform a comprehensive assessment of ovarian aging and immune cell infiltration. An increased plasma cell infiltration and decreased naïve CD4^+^ T cell infiltration are involved in ovarian aging. Pioli et al. (2019) reported that, in mice, plasma cell number was increased in the bone marrow during aging, suggesting a high expression of genes encoding inflammatory cytokines. It has been demonstrated that naïve CD4^+^ T cells from aged animals exhibit age-related phenotypes of immune response; moreover, there are fewer naïve CD4^+^ T cells in the periphery responding to newly encountered antigens, which may affect the immune response of aged individuals (Lefebvre and Haynes, 2012).

Beyond antibody secretion, some long-lived plasma cells produce autoreactive antibodies, which lead to the pathogenesis and development of chronic autoimmune diseases, including lupus erythematosus, rheumatoid arthritis, or multiple sclerosis (Lindquist et al., 2019). Additionally, the senescent mature B cells exhibit limited effective antibody response capabilities, thereby contributing to a chronic low-grade inflammation during aging (Hagen and Derudder, 2020). In elderly people, thymic activity decreases and ceases with age, demonstrating a decline in numbers of naïve T cells. The main characteristics of CD4^+^ T cells during aging include loss of proliferation and decreased telomerase activity, TCR restriction, and low IL-2 and high IFN-γ production and response, which were highly relevant to the inflammatory response showed in Figure 4. However, research on the dynamics between plasma cells, naïve CD4^+^ T cells, and ovarian aging remains limited; further experimental evidence and mechanisms are still needed.

The correlation analysis of hub genes (*Lcp2* and *H2-Ab1*) indicated that they were positively correlated with memory B cells, plasma cells, m1 macrophages, Th17 cells, and immature dendritic cells, whereas *H2-Ab1* was negatively correlated with naïve CD4^+^ T cells, Th2 cells, and resting NK cells. A previous study indicated that memory B cells contained non-functional short telomeres, and the number of memory B cells increased with age (Colonna-Romano et al., 2009). Furthermore, the age-associated macrophage polarization to a pro-inflammatory M1 or anti-inflammatory M2 subtype may dysregulate the development of the host response in the elderly (Mahbub et al., 2012). Aged BALB/c mice showed increased intermuscular adipose tissue and M2 macrophages in skeletal muscle with slightly increased collagen protein production, indicating that the effects of aging on muscle metabolism in aged skeletal muscles may be due to increased M2 macrophages; however, the number of M1 macrophages in skeletal muscles was considerably lower and decreased with age (Cui et al., 2019). In healthy elderly individuals, the frequency of Th17 cells and levels of Th17-related cytokines in peripheral blood were higher than those in young healthy subjects (Lee et al., 2011). Previous studies have confirmed that in elderly subjects, decreased NK cell activity is associated with increased incidence and severity of various age-related diseases such as infectious diseases, cardiovascular disease, liver fibrosis, and cancer (Gounder et al., 2018). Also, as the upregulated DEGs screened out were significantly enriched in immune response and inflammatory response pathway in ovarian aging and the correlation of hub genes and immune cell infiltration, we speculate that ovarian aging is associated with changes in the immune cell frequency and distribution based on our results. The aging of immune system, i.e., “immunosenescence,” which refers to a chronic and low-grade proinflammatory state, has been used to explain ovarian aging (Broekmans et al., 2009). Shirasuna and Iwata (2017) reported that cellular senescence and inflammaging could accelerate reproductive failure by promoting senescence-associated secretory phenotype and immunosenescence during pregnancy. From our immune cell infiltration cell analysis, we could suggest that an increased plasma cell infiltration and decreased naïve CD4 + T cell infiltration indicated the phenotype of immunosenescence, which could explain the mechanisms of aging in the ovary to some extent.

However, more concrete evidence is urgently needed to explain the complexity of ovarian aging and the involvement of transcriptional changes and immune cell network. On the other hand, we identified other pathways like MYC and mTORC1 pathways with the lowest *t* value of GSVA score in GSVA results. *Myc* regulates gene transcription and appears to promote cellular growth and has been linked to aging-related genes such as WRN (Grandori et al., 2003) and TERT (Wu et al., 1999). As a nutrient sensor, it has been largely acknowledged that the mTOR pathway is involved in regulating lifespan and aging (Papadopoli et al., 2019). The inhibition of mTOR signaling can extend ovarian lifespan, with the evidence that rapamycin can increase the number of primordial follicles in rats (Guo and Yu, 2019).

To date, there is little research on the genes involved in ovarian aging. In the present study, we used transcriptional data analysis to deduce the various pathways associated with ovarian aging. The qPCR results confirmed that the hub genes *Tyrobp*, *Rac2*, *Cd14*, *Zap70*, *Lcp2*, *Itgb2*, *H2-ab1*, and *Fcer1g* were all upregulated as suggested by the bioinformatic analysis. Moreover, specific antibodies to proteins encoded by the highly significant hub genes – LCP2, ITGB2, and CD14 – were more highly expressed in the ovaries of old mice than young mice. Combining our results and the above description, we speculate that the top hub genes listed may serve as the potential biomarkers of ovarian aging. Nevertheless, the above analysis and verification were based on mice models and may not translate into human trials. Considering the complex etiology and mechanisms of ovarian aging, further research should be performed to reinforce the findings of the present study.

## Conclusion

The transcriptomic analysis showed different gene expression patterns in the ovaries of young and old mice. The DEGs involved in ovarian aging are highly associated with the BP and pathways related to the immune system, cell–CAMs, and phagosomes. In addition, the hub genes identified were further confirmed to be expressed in the ovaries of old and young mice. Thus, we speculated that the immune system plays a pivotal role in ovarian aging. Future studies should explore the potential targets for delaying ovarian aging on the basis of the above findings. However, related genes in human ovarian tissue remain to be verified, and further research is needed to identify the genetic and molecular mechanisms underlying ovarian aging.

## Data Availability Statement

The original contributions presented in the study are included in the article/Supplementary Material, further inquiries can be directed to the corresponding author/s.

## Ethics Statement

The animal study was reviewed and approved by Tongji Hospital, Tongji Medical College, Huazhong University of Science and Technology in China.

## Author Contributions

SW and JZ conceptualized and coordinated the study. SW and LM designed the experiments. LM and HL conducted all the major experiments, visualized the data, and wrote the manuscript. RC, MW, and YJ performed the experiments and analyzed the data. All the authors have read and approved the final version of manuscript.

## Conflict of Interest

The authors declare that the research was conducted in the absence of any commercial or financial relationships that could be construed as a potential conflict of interest.

## Footnotes

1 www.ncbi.nlm.nih.gov/geo/

2 http://bioconductor.org/

3 http://geneontology.org/

## Supplementary Material

Supplementary Figure 1 A global view of GO functional classification and clustering by calculating the Kappa value with DAVID algorithms.

Supplementary Table 1 The list of differentially expressed genes.

Supplementary Table 2 The list of KEGG pathways.
