# Supplementary figures and images for: Identification of Key Genes and Potential New Biomarkers for Ovarian Aging: A Study Based on RNA-Sequencing Data
Source: Front Genet. 2020 Nov 16;11:590660. doi: 10.3389/fgene.2020.590660 (PMC7701310; doi:10.3389/fgene.2020.590660)

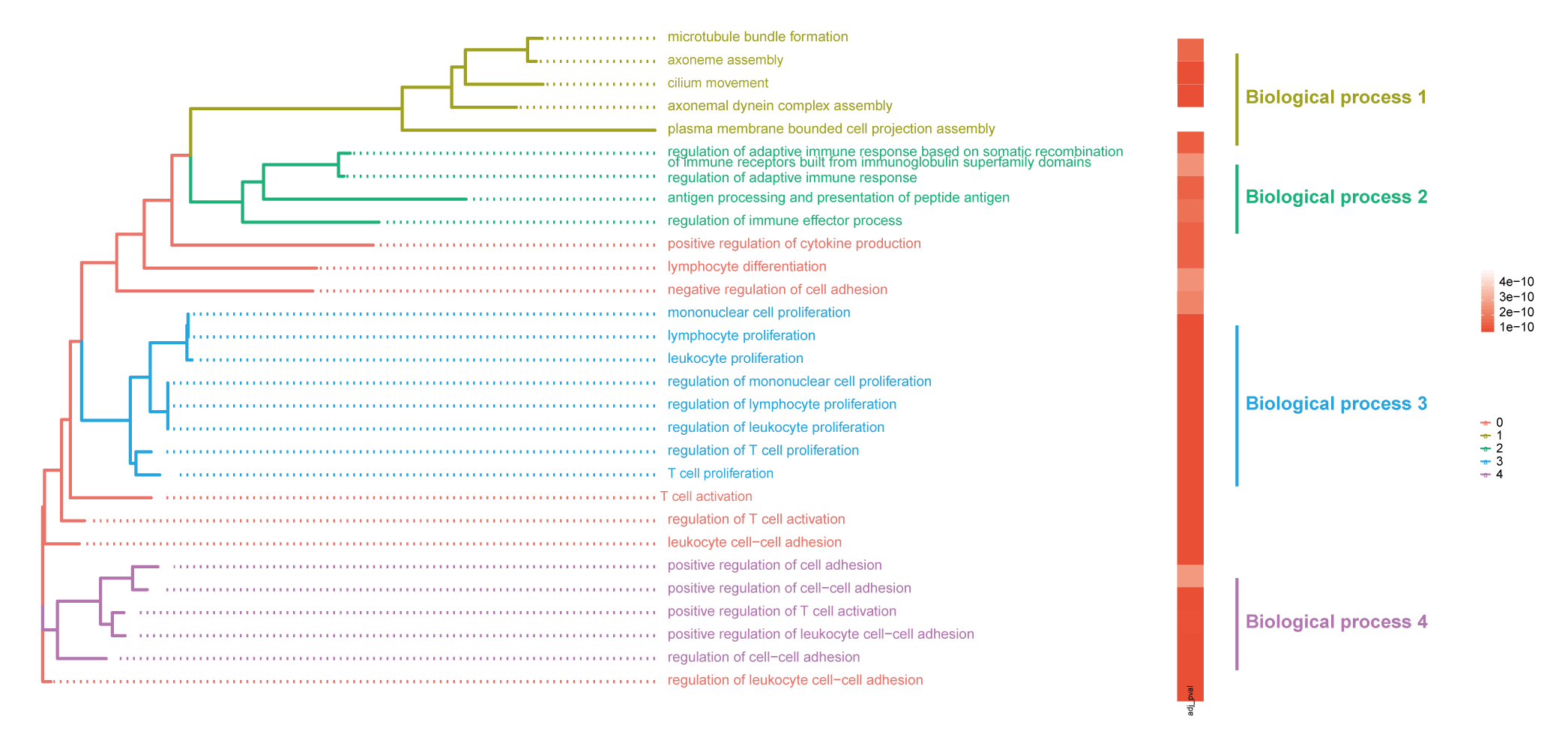

Supplement: Supplementary Figure 1 — A global view of GO functional classification and clustering by calculating the Kappa value with DAVID algorithms. [file Image_1.TIF]
