# Supplementary material for: Identification of Key Genes and Potential New Biomarkers for Ovarian Aging: A Study Based on RNA-Sequencing Data
Source: Front Genet. 2020 Nov 16;11:590660. doi: 10.3389/fgene.2020.590660 (PMC7701310; doi:10.3389/fgene.2020.590660)
Supplement: Supplementary Table 2 — The list of KEGG pathways. [file Table_2.DOCX]

| ID | Description | GeneRatio | pvalue | p.adjust | geneID |
| --- | --- | --- | --- | --- | --- |
| mmu04514 | Cell adhesion molecules (CAMs) | 41/356 | 7.49E-21 | 1.95E-18 | 242122/12738/58187/12739/53624/12740/12550/16408/11658/12481/19264/16728/  100504404/14999/75677/15006/14969/20737/269116/14961/14960/12504/16414/  667977/16421/14998/16409/15007/12483/18260/15001/60363/15015/14990/60533/  14972/667803/17528/20612/15013/15894 |
| mmu04145 | Phagosome | 30/356 | 3.83E-11 | 4.99E-09 | 20390/12266/140494/242341/100504404/14999/13040/15006/56644/1972/14969/12721/  14961/14960/12475/13058/16414/667977/24088/14998/16409/15007/14129/15001/  15015/14990/14972/667803/15013/21354 |
| mmu04640 | Hematopoietic cell lineage | 21/356 | 1.65E-10 | 1.44E-08 | 12985/12478/12484/12481/12493/100504404/14999/12502/14969/14961/14960/12504/  12475/12507/12501/14998/16409/16790/14129/12483/15001 |
| mmu05416 | Viral myocarditis | 20/356 | 2.68E-10 | 1.75E-08 | 16408/100504404/14999/15006/14969/14961/14960/18646/16414/19354/667977/14998/  15007/15001/15015/14990/14972/667803/15013/15894 |
| mmu05330 | Allograft rejection | 17/356 | 3.39E-10 | 1.77E-08 | 100504404/14999/15006/14969/14961/14960/18646/667977/14998/15007/15001/15015/  14990/14972/667803/15013/14102 |
| mmu04612 | Antigen processing and presentation | 20/356 | 5.09E-10 | 2.16E-08 | 100504404/14999/13040/15006/14969/14961/16149/14960/12504/12265/667977/14998/  15007/15001/15015/14990/14972/667803/15013/21354 |
| mmu05332 | Graft-versus-host disease | 17/356 | 5.79E-10 | 2.16E-08 | 100504404/14999/15006/14969/14961/14960/18646/667977/14998/15007/15001/15015/  14990/14972/667803/15013/14102 |
| mmu05169 | Epstein-Barr virus infection | 32/356 | 9.18E-10 | 3.00E-08 | 215446/12478/16408/100504404/14999/23960/12502/15006/14969/15945/17060/14961/  14960/26415/667977/12503/21929/12501/24088/14998/15007/29857/15001/15015/  14990/14972/667803/246730/15013/14102/15894/21354 |
| mmu05140 | Leishmaniasis | 17/356 | 1.59E-09 | 4.29E-08 | 12266/100504404/14999/17972/14969/14961/14960/13058/16414/26415/15170/24088/  14998/16409/29857/14129/15001 |
| mmu05320 | Autoimmune thyroid disease | 18/356 | 1.64E-09 | 4.29E-08 | 22095/100504404/14999/15006/14969/14961/14960/18646/667977/14998/15007/15001/  15015/14990/14972/667803/15013/14102 |
| mmu04940 | Type I diabetes mellitus | 17/356 | 2.02E-09 | 4.80E-08 | 100504404/14999/15006/14969/14961/14960/18646/667977/14998/15007/15001/15015/  14990/14972/667803/15013/14102 |
| mmu05152 | Tuberculosis | 27/356 | 3.27E-09 | 7.12E-08 | 12266/16803/140494/242341/100504404/14999/13040/56644/1499/12721/14961/16149/  14960/12475/16414/12265/26415/24088/14998/16409/29857/14129/15001/75600/16154  /12323/14127 |
| mmu05150 | Staphylococcus aureus infection | 20/356 | 1.02E-07 | 2.04E-06 | 12266/16668/16408/12630/16669/100504404/14999/14962/14969/14961/14960/12268/  16414/14998/16409/94179/19204/14129/15001/15894 |
| mmu04672 | Intestinal immune network for IgA production | 11/356 | 6.63E-07 | 1.24E-05 | 56838/72049/100504404/14999/14969/14961/14960/18703/16421/14998/15001 |
| mmu04659 | Th17 cell differentiation | 17/356 | 7.38E-07 | 1.28E-05 | 100504404/14999/16797/12502/14969/14961/14960/12504/60504/26415/12503/12501/  16364/14998/22637/29857/15001 |
| mmu04670 | Leukocyte transendothelial migration | 18/356 | 9.09E-07 | 1.48E-05 | 12738/58187/12739/53624/12740/16408/75677/17972/13058/16414/26415/19354/  16409/29857/18260/60363/22350/15894 |
| mmu05340 | Primary immunodeficiency | 10/356 | 1.19E-06 | 1.83E-05 | 12478/12518/19264/72049/17060/12504/12265/12501/22637/21354 |
| mmu05145 | Toxoplasmosis | 17/356 | 1.69E-06 | 2.45E-05 | 16780/16782/100504404/14999/14969/14961/14960/12774/12265/26415/24088/14998/  29857/15001/16154/16776/16774 |
| mmu05323 | Rheumatoid arthritis | 15/356 | 1.84E-06 | 2.53E-05 | 16408/140494/16994/242341/100504404/20304/14999/14969/14961/14960/  16414/24088/14998/15001/15894 |
| mmu04658 | Th1 and Th2 cell differentiation | 15/356 | 2.15E-06 | 2.80E-05 | 100504404/14999/16797/12502/14969/14961/14960/12504/26415/12503/  12501/14998/22637/29857/15001 |
| mmu05310 | Asthma | 8/356 | 3.12E-06 | 3.88E-05 | 100504404/14999/14969/14961/14960/14998/15001/14127 |
| mmu04662 | B cell receptor signaling pathway | 14/356 | 5.29E-06 | 6.28E-05 | 12478/12518/15985/18726/83490/14728/68713/17060/19354/18733/15170/  12483/12517/108723 |
| mmu04530 | Tight junction | 20/356 | 1.44E-05 | 0.000164 | 72058/27375/12738/58187/12739/53624/12740/71960/269643/73608/23859/  72930/75677/12638/70737/18260/60363/22350/15163/18762 |
| mmu04650 | Natural killer cell mediated cytotoxicity | 16/356 | 2.17E-05 | 0.000236 | 16408/16797/18646/16414/19354/15170/12503/22637/23900/14972/22035/  16822/22177/14102/14127/15894 |
| mmu05166 | Human T-cell leukemia virus 1 infection | 25/356 | 2.55E-05 | 0.000266 | 16408/72049/100504404/14999/12502/15006/14969/14961/14960/12504/16414/  667977/12501/12578/14998/20375/15007/15001/15015/14990/17701/14972/  667803/15013/15894 |
| mmu05164 | Influenza A | 19/356 | 4.86E-05 | 0.000488 | 50528/100504404/20304/14999/23960/14969/15945/14961/14960/12265/76815/  17229/14998/15001/22035/246730/58185/14102/15894 |
| mmu05321 | Inflammatory bowel disease (IBD) | 10/356 | 0.000146 | 0.001416 | 100504404/14999/14969/14961/14960/60504/16182/24088/14998/15001 |
| mmu05165 | Human papillomavirus infection | 30/356 | 0.00018 | 0.001677 | 93735/22411/16780/269643/22421/140494/242341/23859/16782/72930/15006/  192897/16420/667977/16421/14368/22422/15007/15015/14990/19219/14972/  16776/667803/23962/18762/16774/15013/14102/22414 |
| mmu04390 | Hippo signaling pathway | 17/356 | 0.000189 | 0.001699 | 93735/22411/14164/12550/22062/269643/22421/23859/72930/211652/16414/14368/  22422/18787/241324/18762/22414 |
| mmu05235 | PD-L1 expression and PD-1 checkpoint pathway in cancer | 12/356 | 0.000238 | 0.002067 | 16797/12502/12504/19419/26415/15170/12503/12501/24088/22637/29857/60533 |
| mmu05142 | Chagas disease (American trypanosomiasis) | 13/356 | 0.000262 | 0.002209 | 12266/269643/20304/72930/12502/26415/12503/12501/24088/29857/18787/14680/14102 |
| mmu04660 | T cell receptor signaling pathway | 13/356 | 0.000289 | 0.00236 | 19264/16797/12502/12504/19419/26415/15170/12503/12501/22637/29857/108723/16822 |
| mmu05170 | Human immunodeficiency virus 1 infection | 22/356 | 0.00031 | 0.00245 | 11768/12502/15006/12504/12774/26415/19354/667977/12503/12501/24088/15007/  29857/15015/14990/75600/14972/667803/108012/15013/14102/21354 |
| mmu04060 | Cytokine-cytokine receptor interaction | 25/356 | 0.000489 | 0.003757 | 12985/50905/57890/56838/16994/72049/232983/20304/230828/213208/15945/12504/  60504/12774/16182/16878/20305/12983/16154/22035/242700/14102/16170/16181/12766 |
| mmu05160 | Hepatitis C | 16/356 | 0.000875 | 0.006522 | 12738/58187/12739/53624/12740/269643/72930/23960/75677/15945/18260/15957/60363/  246730/58185/14102 |
| mmu05134 | Legionellosis | 8/356 | 0.002981 | 0.02161 | 12266/17951/12475/16414/17948/268973/24088/16409 |
| mmu04061 | Viral protein interaction with cytokine and cytokine receptor | 11/356 | 0.003279 | 0.023129 | 56838/20304/230828/213208/15945/12774/16182/20305/16154/22035/12766 |
| mmu04950 | Maturity onset diabetes of the young | 5/356 | 0.004372 | 0.030027 | 21410/15376/18770/15378/17341 |
| mmu05205 | Proteoglycans in cancer | 17/356 | 0.004594 | 0.030744 | 93735/22411/22421/13867/74055/26415/15170/13982/24088/14368/22422/29857/  22350/12323/15163/14102/22414 |
| mmu04610 | Complement and coagulation cascades | 10/356 | 0.004726 | 0.030839 | 12266/12630/14962/12268/20701/16414/14066/16409/14058/18787 |
| mmu04064 | NF-kappa B signaling pathway | 11/356 | 0.005432 | 0.034582 | 16803/16994/72049/16797/17060/12475/21929/22637/22029/108723/15894 |
